# In Vitro and In Vivo Efficacy of the Broad-Spectrum β-Lactamase Inhibitor APC24-7 in Combination with Meropenem Against Carbapenem-Resistant *Enterobacterales*

**DOI:** 10.3390/antibiotics15101011

**Published:** 2026-10-12

**Authors:** Carina Sofia Silva Matias, Klaus Skovbo Jensen, Henrik Hasman, Kasper Thystrup Karstensen, Bjørg Bolstad, Bjørn Klem, Pål Rongved, Carina Vingsbo Lundberg, Jon Ulf Hansen

**Affiliations:** 1 Bacteria, Parasites & Fungi, Statens Serum Institut (SSI), 2300 Copenhagen, Denmark; henh@ssi.dk (H.H.); ktka@ssi.dk (K.T.K.); cvl@ssi.dk (C.V.L.); joa@ssi.dk (J.U.H.); 2 CANDOR Simulation, 8270 Højbjerg, Denmark; ksj@candorsim.dk; 3 AdjuTec Pharma AS, 0349 Oslo, Norway; bb@adjutecpharma.com (B.B.); bk@adjutecpharma.com (B.K.); pr@adjutecpharma.com (P.R.); 4 Department of Pharmacy, The University of Oslo, 0371 Oslo, Norway

**Keywords:** APC24-7, meropenem, β-lactamase inhibitor, combination therapy, carbapenem-resistant *Enterobacterales*, metallo-β-lactamases, serine β-lactamases, in vivo infection model, pharmacokinetics

## Abstract

Background: Carbapenem-resistant *Enterobacterales* (CRE) represent a major therapeutic challenge, driven in part by serine β-lactamases (SBLs) and metallo-β-lactamases (MBLs). APC24-7 is a broad-spectrum β-lactamase inhibitor (BLI) designed to inhibit enzymes from both groups. Here, we evaluated the ability of APC24-7 to potentiate meropenem (MEM) activity against meropenem-resistant *Escherichia coli* and *Klebsiella pneumoniae*. Methods & Results: Checkerboard assays were performed against 31 *K. pneumoniae* and 17 *E. coli* isolates harboring a range of clinically relevant SBL and/or MBL genes. APC24-7 reduced meropenem minimum inhibitory concentrations (MICs) in an isolate- and concentration-dependent manner. At 32 mg/L APC24-7, meropenem MICs fell within the CLSI susceptible range for meropenem in 11 of 17 *E. coli* isolates, whereas 16 of 31 *K. pneumoniae* isolates remained resistant. Time-kill experiments with nine selected isolates showed bactericidal activity under active combination conditions, defined as a ≥3 log10 CFU/mL reduction from the initial bacterial count, within 3–5 h. Pharmacokinetic evaluation in infected mice showed less-than-dose-proportional systemic exposure following subcutaneous administration of APC24-7, with a maximum plasma concentration of approximately 26 mg/L after a 30 mg/kg dose. In a neutropenic murine thigh infection model, APC24-7 increased meropenem activity against four selected in vitro-responsive isolates in a dose-dependent manner. Conclusions: These findings provide preclinical proof of concept for APC24-7 as a broad-spectrum BLI capable of potentiating meropenem against *Enterobacterales* harboring diverse β-lactamases, while highlighting substantial isolate-dependent variability that warrants further pharmacokinetic/pharmacodynamic and resistance-mechanism studies.

## 1. Introduction

The emergence and global spread of multidrug-resistant bacteria constitute a major public health threat. Carbapenem-resistant *Enterobacterales* (CRE), which include clinically important species such as *Escherichia coli* and *Klebsiella pneumoniae*, are designated critical-priority bacterial pathogens by the World Health Organization (WHO) and are associated with high rates of antibiotic treatment failure and mortality [1,2,3]. β-lactam antibiotics (BLs), including carbapenems, remain among the most widely used agents in hospital settings because of their efficacy and broad spectrum of activity [1,2]. However, increasing resistance threatens their continued effectiveness and highlights the need for new treatment strategies [4,5]. Resistance to BLs arises through several mechanisms, among which β-lactamase production is one of the most clinically important. β-lactamases can be classified as zinc-dependent metallo-β-lactamases (MBLs; Ambler class B) or nucleophilic serine β-lactamases (SBLs; Ambler classes A, C, and D) [6,7,8]. Enzymes belonging to classes A, B, and D are frequently associated with carbapenem resistance [9]. MBLs and SBLs hydrolyze the β-lactam ring through distinct mechanisms and differ in their substrate specificity and distribution among bacterial species [7,10]. Multiple β-lactamases may coexist with additional resistance mechanisms; consequently, CRE are often resistant to several antimicrobial classes, substantially limiting treatment options [11,12,13].

Significant advances have been made in the development of novel β-lactamase inhibitors (BLI*s*) [4,14,15]. BLIs are used in combination with existing BLs to protect the partner antibiotic from enzymatic degradation, thereby restoring or enhancing antibacterial activity [16,17]. Since 2015, six novel BL/BLI combinations, aztreonam-avibactam [18,19], ceftazidime-avibactam (CZA), meropenem-vaborbactam [20], imipenem-relebactam (IMR) [21], sulbactam/durlobactam [22] and cefepime-enmetazobactam [23], have been approved, providing additional treatment options for infections caused by CRE. Most of these inhibitors primarily target SBL-mediated resistance, particularly class A and C enzymes, and have limited activity against class D enzymes; durlobactam is an important exception with potent activity against class D β-lactamases [15,24].

Infections caused by MBL-producing pathogens remain difficult to treat [15]. Current options include aztreonam combined with avibactam or ceftazidime-avibactam, as well as monotherapy with the novel siderophore cephalosporin cefiderocol [25,26,27,28,29]. Although cefiderocol is active against MBL-producing organisms, resistance has already been reported [30,31,32,33].

To date, no direct MBL inhibitor has received clinical approval; however, several promising compounds are at different stages of development, with some having reached Phase 3 clinical evaluation [4,14,15,34]. Direct MBL inhibitors described to date include ANT2681 [35] and indole carboxylate inhibitors [36]. In addition, BL/BLI combinations, such as β-lactam/APC148 [37], cefepime/taniborbactam [38], and β-lactam/xeruborbactam (QPX7728) [37], entered clinical evaluation and provide activity against both SBL- and MBL-producing organisms through direct inhibition or complementary combination strategies. Other approaches provide activity against MBL-producing organisms without directly inhibiting MBLs. Notably, FDA-approved cefepime/zidebactam (WCK 5222; Zaynich), in which zidebactam combines β-lactamase inhibition with high-affinity PBP binding and enhancer activity, was recently approved for the treatment of complicated urinary tract infections, including pyelonephritis [39,40]. Cefepime/nacubactam similarly combines β-lactamase inhibition with PBP-binding/enhancer activity and remains under clinical development [41,42]. Thus, whereas zidebactam and nacubactam can provide activity against MBL-producing organisms through β-lactam enhancer activity, compounds such as taniborbactam and APC24-7 are designed to directly inhibit MBLs.

APC24-7 was recently described as a hybrid broad-spectrum BLI that combines boronate-based inhibition with a metal-chelating moiety, thereby providing activity against both SBLs and MBLs. Previous characterization showed that APC24-7 reduced meropenem MICs to or below the meropenem susceptible breakpoint in isolates harboring clinically relevant β-lactamases and showed greater activity against NDM-9 and IMP-26 than taniborbactam, whereas activity against OXA-48 was limited [43]. These findings distinguish APC24-7 from other broad-spectrum BLIs such as taniborbactam, for which reduced activity against certain NDM and IMP variants has been reported. However, the activity of APC24-7 against a broader panel of genetically diverse clinical isolates and its efficacy in an in vivo infection model remain incompletely characterized.

In the present study, we evaluated APC24-7 in combination with meropenem against a diverse panel of meropenem-resistant *K. pneumoniae* and *E. coli* isolates harboring clinically relevant SBL and/or MBL genes. We characterized the concentration-dependent effect of APC24-7 on meropenem MICs, assessed bacterial killing using time-kill experiments and pharmacodynamic modeling, determined the pharmacokinetic profile of APC24-7 in infected mice, and evaluated the combination in a neutropenic murine thigh infection model. Together, these experiments extend the previous mechanistic characterization of APC24-7 and provide preclinical evidence of activity against genetically diverse isolates, including in vivo proof of concept.

## 2. Results

### 2.1. APC24-7 Potentiates Meropenem Activity

**Figure 1:**
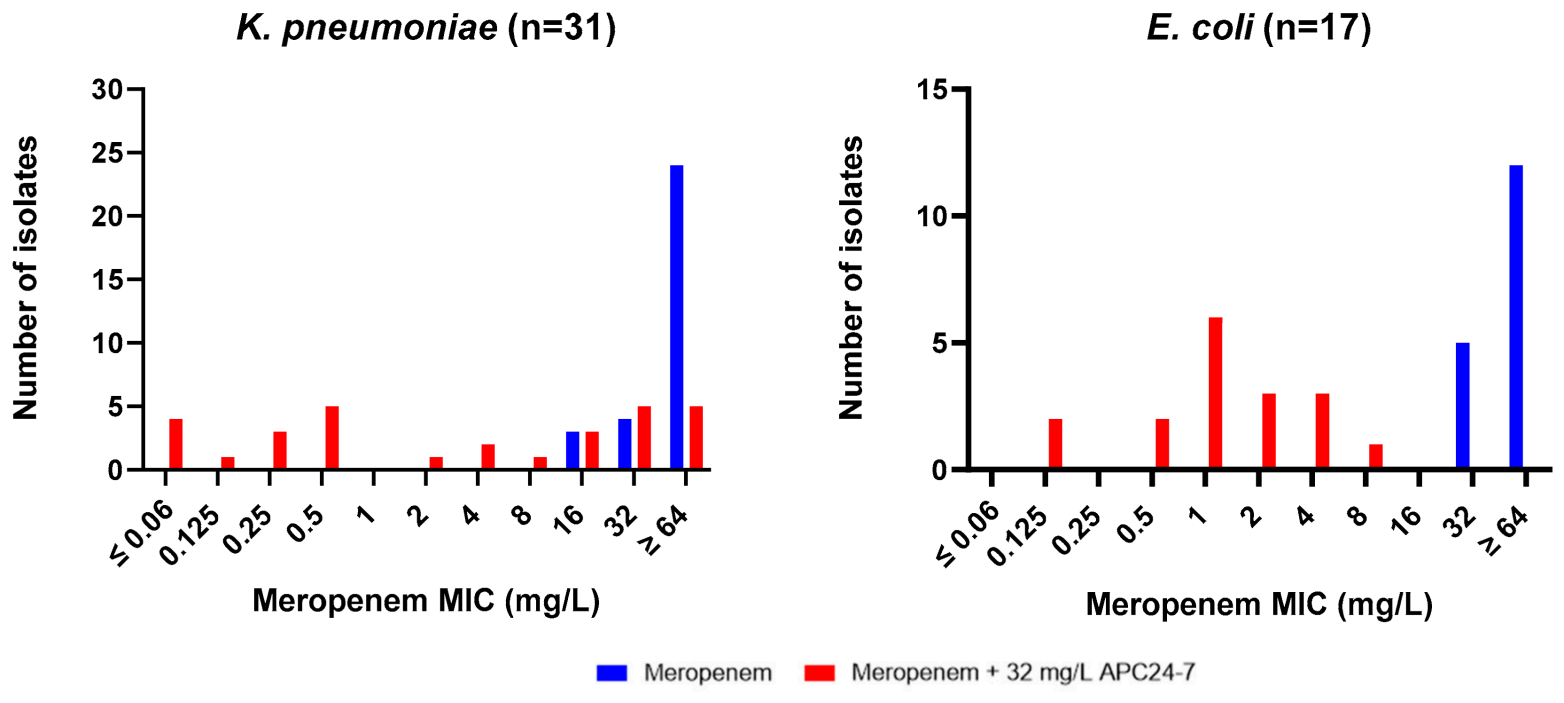
Distribution of meropenem MICs alone and in combination with 32 mg/L APC24-7 for *K. pneumoniae* (*n* = 31) and *E. coli* (*n* = 17). Blue bars indicate meropenem alone; red bars indicate meropenem combined with 32 mg/L APC24-7. APC24-7 shifted the meropenem MIC distribution toward lower values.

The MICs of meropenem and APC24-7 were determined for a panel of 48 clinical and reference *K. pneumoniae* and *E. coli* isolates harboring a broad range of SBL and/or MBL genes. All isolates were classified as resistant to meropenem (MIC ≥ 4 mg/L). APC24-7 showed no intrinsic antibacterial activity at concentrations up to 64 mg/L.. To assess the ability of APC24-7 to potentiate meropenem activity, checkerboard assays were performed with increasing APC24-7 concentrations in combination with meropenem against 31 *K. pneumoniae* strains (Table 1) and 17 *E. coli* strains (Table 2). APC24-7 reduced meropenem MICs in a subset of isolates in a concentration-dependent manner. Potentiation was observed across multiple isolates, with meropenem MICs reaching the CLSI susceptible range (MIC ≤ 1 mg/L) [44] at APC24-7 concentrations as low as 4–8 mg/L. Figure 1 summarizes the checkerboard assay results at an APC24-7 concentration of 32 mg/L. At this concentration, meropenem MICs fell within the CLSI susceptible range for meropenem in 11 of 17 *E. coli* isolates, including isolates harboring OXA-48 or NDM-5 β-lactamase genes. In contrast, 16 of 31 *K. pneumoniae* isolates remained resistant to meropenem.

### 2.2. Killing Kinetics of Meropenem in Combination with APC24-7

To further evaluate the activity of meropenem in combination with APC24-7, time-kill studies were performed using five meropenem-resistant *K. pneumoniae* isolates and four meropenem-resistant *E. coli* isolates (MIC ≥ 32 mg/L). Isolates were selected based on their responsiveness to the meropenem/APC24-7 combination in checkerboard assays at APC24-7 concentrations of 64 mg/L or lower and on the presence of clinically relevant MBL and/or SBL genes, including NDM, VIM, IMP, OXA, and KPC.

Bacterial cultures were exposed for 24 h to varying concentrations of meropenem combined with a fixed APC24-7 concentration of 32 mg/L. Meropenem monotherapy controls were included for all isolates and are shown in Figure S1.

**Figure 2:**
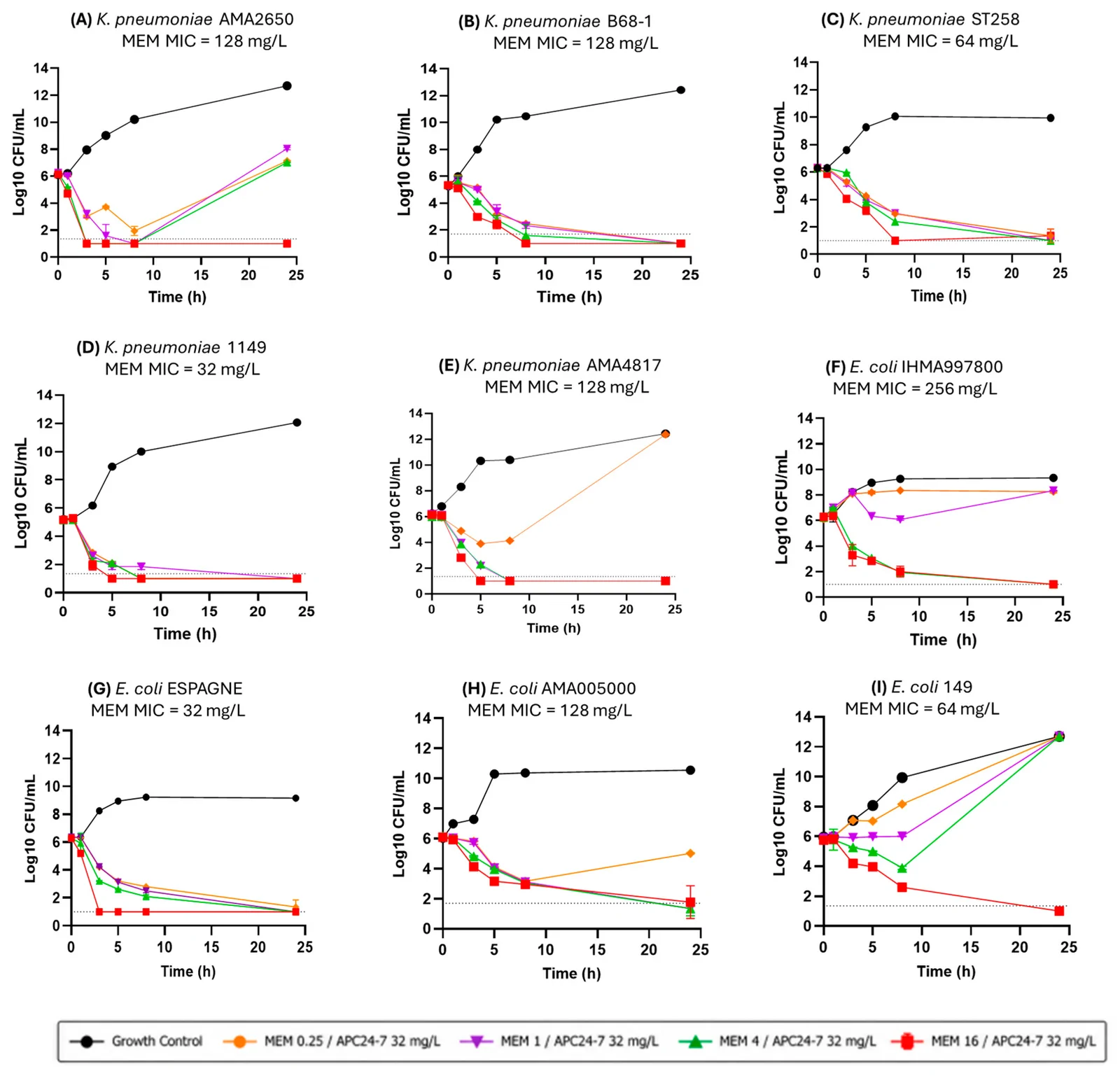
Time-kill curves for meropenem-resistant clinical isolates (MEM MIC ≥ 32 mg/L): (**A**) *K. pneumoniae* AMA2650, (**B**) *K. pneumoniae* B68-1, (**C**) *K. pneumoniae* ST258, (**D**) *K. pneumoniae* 1149, (**E**) *K. pneumoniae* AMA4817, (**F**) *E. coli* IHMA997800, (**G**) *E. coli* Espagne, (**H**) *E. coli* AMA005000, and (**I**) *E. coli* 149. Isolates were treated with meropenem at 0.25, 1, 4, or 16 mg/L in combination with a fixed APC24-7 concentration of 32 mg/L. Growth controls (drug-free medium) were included in all experiments. The dotted line in each graph indicates the lower limit of detection. Data are shown as +mean ± SD log_10_ CFU/mL from duplicate measurements at each time point.

In combination with 32 mg/L APC24-7, meropenem produced bactericidal activity, defined as a ≥3 log10 CFU/mL reduction from the initial bacterial count [45], within 3–5 h for all nine selected isolates (Figure 2). For the five *K. pneumoniae* isolates, 1 mg/L meropenem was sufficient to achieve bactericidal activity in combination with APC24-7, whereas 1–16 mg/L meropenem was required for the *E. coli* isolates. Comparison with meropenem monotherapy (Figure S1) showed that bactericidal activity occurred with the combination rather than with meropenem alone.

Regrowth at later time points occurred predominantly with meropenem monotherapy and less frequently under active combination conditions. Preliminary susceptibility testing of selected colonies recovered during regrowth showed increased meropenem MICs that persisted after growth in drug-free medium, suggesting possible selection of less susceptible subpopulations. Further characterization of these populations is required.

### 2.3. Killing Rates of Meropenem in Combination with APC24-7

**Figure 3:**
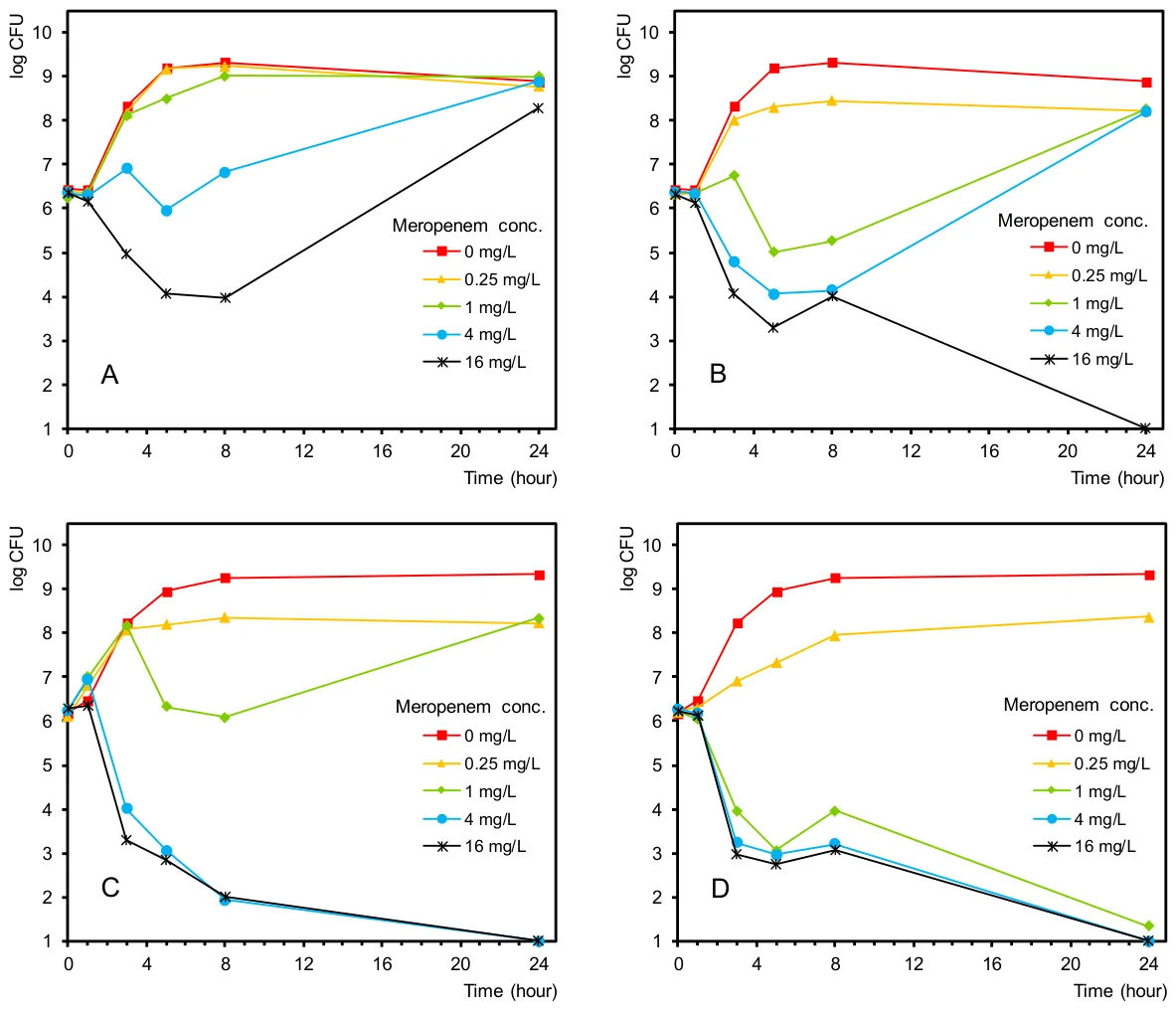
Time-kill curves for *E. coli* IHMA997800 treated with meropenem at 0.25, 1, 4, or 16 mg/L in combination with APC24-7 at (**A**) 2, (**B**) 8, (**C**) 32, or (**D**) 128 mg/L.

**Figure 4:**
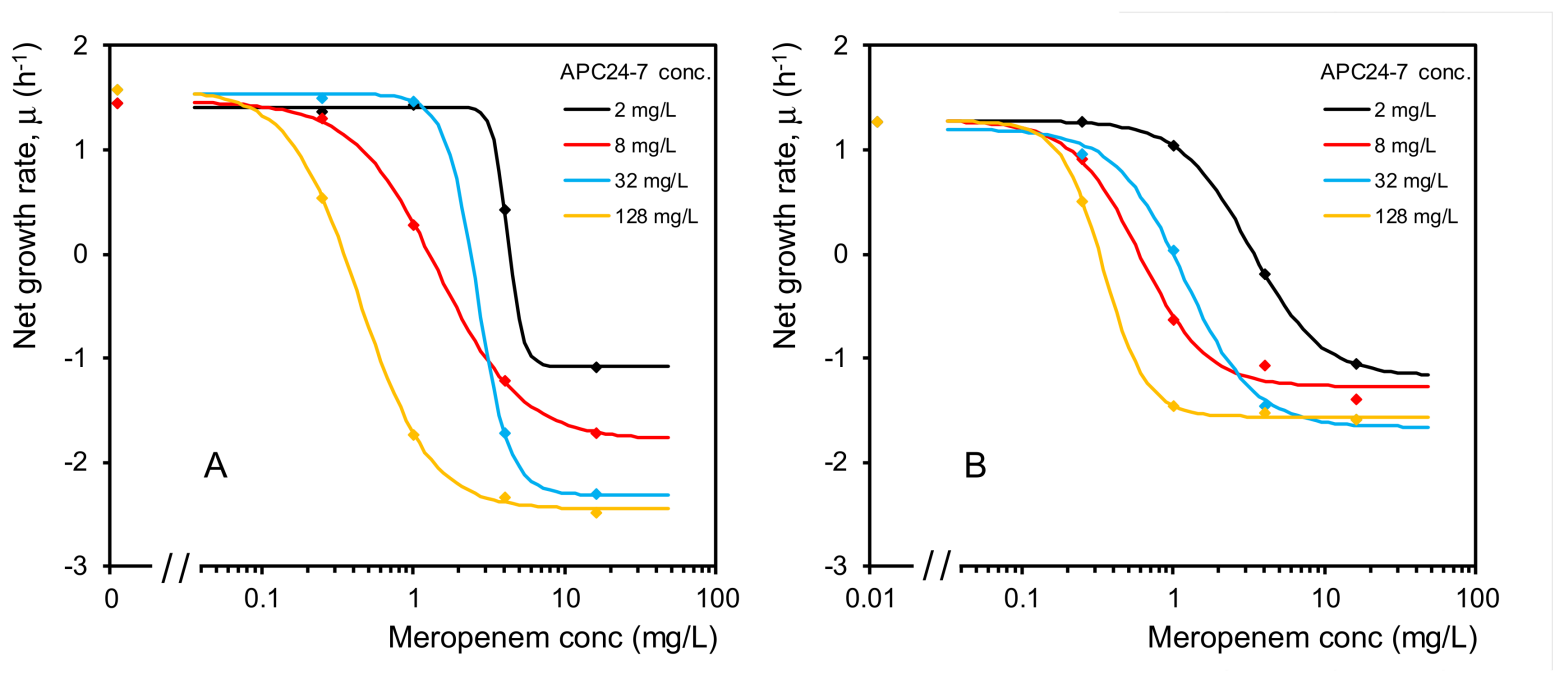
Initial net growth rate of *E. coli* IHMA997800 for different combinations of meropenem and APC24-7 concentrations. The net growth rate is shown as the mean over t = 0–3 h (**A**) and t = 0–5 h (**B**) of the time-kill experiments.

To further characterize the combined effects of APC24-7 and meropenem on bacterial growth, four sets of time-kill curves for E. coli IHMA997800 were analyzed using a two-dimensional approach (Figure 3). This analysis estimated the initial net growth rate as a function of the simultaneous concentrations of meropenem and APC24-7 (Figure 4). APC24-7 concentration had a pronounced effect on the meropenem EC_50_ and on the shape of the fitted sigmoid concentration-response curve. The pharmacodynamic MIC (zMIC), the zero-net-growth MIC, defined as the meropenem concentration at which µ = 0, is closely related to EC_50_. Using the initial 3 h interval, zMIC decreased from 4.3 to 0.35 mg/L as the APC24-7 concentration increased from 2 to 128 mg/L (Figure 4A). When the initial 5 h interval was used, zMIC decreased from 3.4 to 0.32 mg/L over the same APC24-7 concentration range (Figure 4B).

### 2.4. APC24-7 Pharmacokinetics

**Figure 5:**
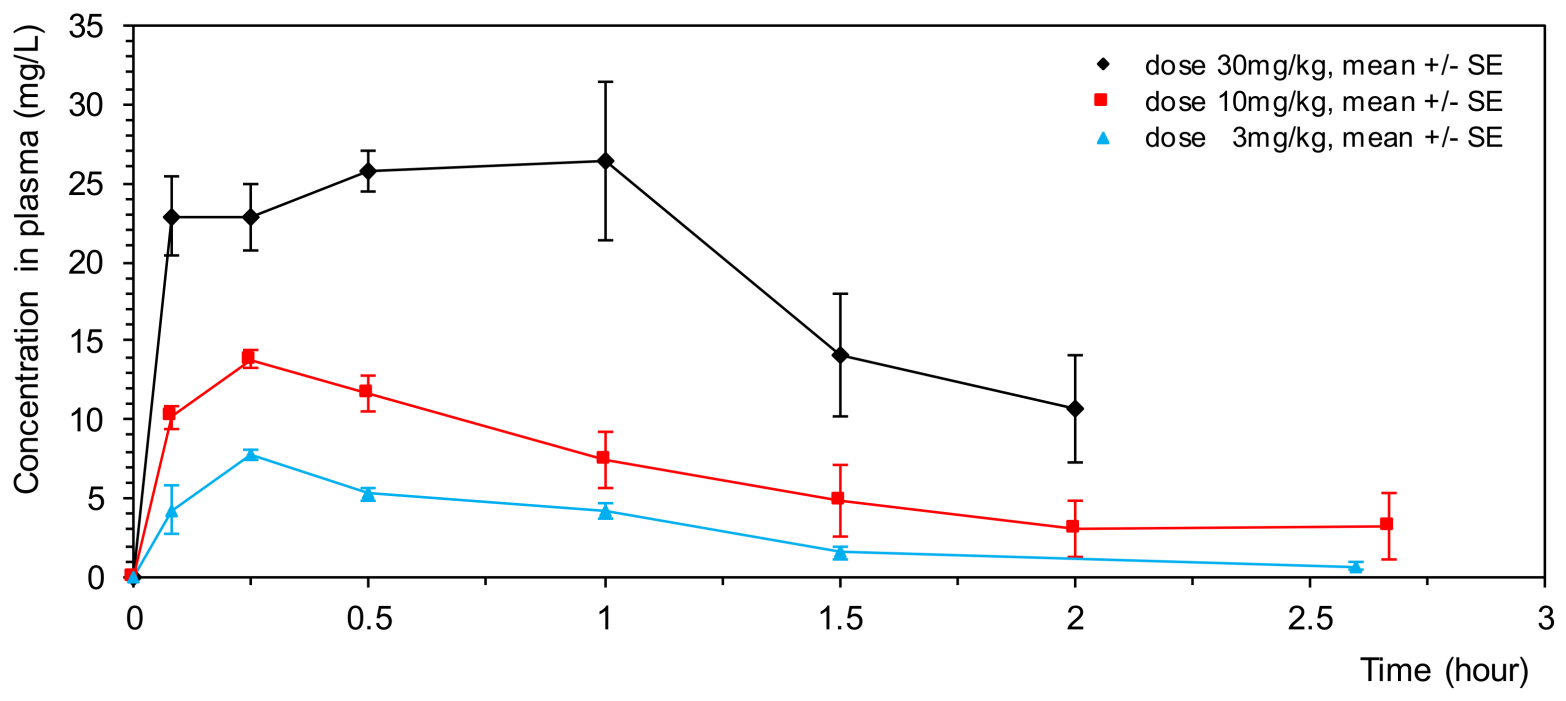
Plasma APC24-7 concentrations following a subcutaneous bolus dose in thigh-infected female NMRI mice.

Following SC administration, systemic APC24-7 exposure increased with dose but less than proportionally over the 3–30 mg/kg range (Figure 5). Both dose-normalized C_max_ and AUC_inf_ decreased with increasing dose, and T_max_ was delayed at 30 mg/kg compared with the lower doses. Because only subcutaneous (SC) administration was evaluated, these data cannot distinguish dose-dependent absorption or bioavailability from nonlinear systemic disposition. The apparent terminal half-life following SC administration was approximately 0.7–0.8 h. At 30 mg/kg, the observed C_max_ was approximately 26 mg/L and AUC_inf_ was approximately 51 h·mg/L (Table 3).

### 2.5. APC24-7 Increases the In Vivo Efficacy of Meropenem

The efficacy of APC24-7 in combination with meropenem was evaluated in a neutropenic murine thigh infection model using four isolates: *E. coli* Espagne, *E. coli* AMA005000, *K. pneumoniae* AMA2650, and *K. pneumoniae* ST258. These isolates were selected based on their in vitro response to APC24-7, β-lactamase diversity, ability to establish infection in vivo, and limited response to meropenem monotherapy in preliminary experiments ().

**Figure 6:**
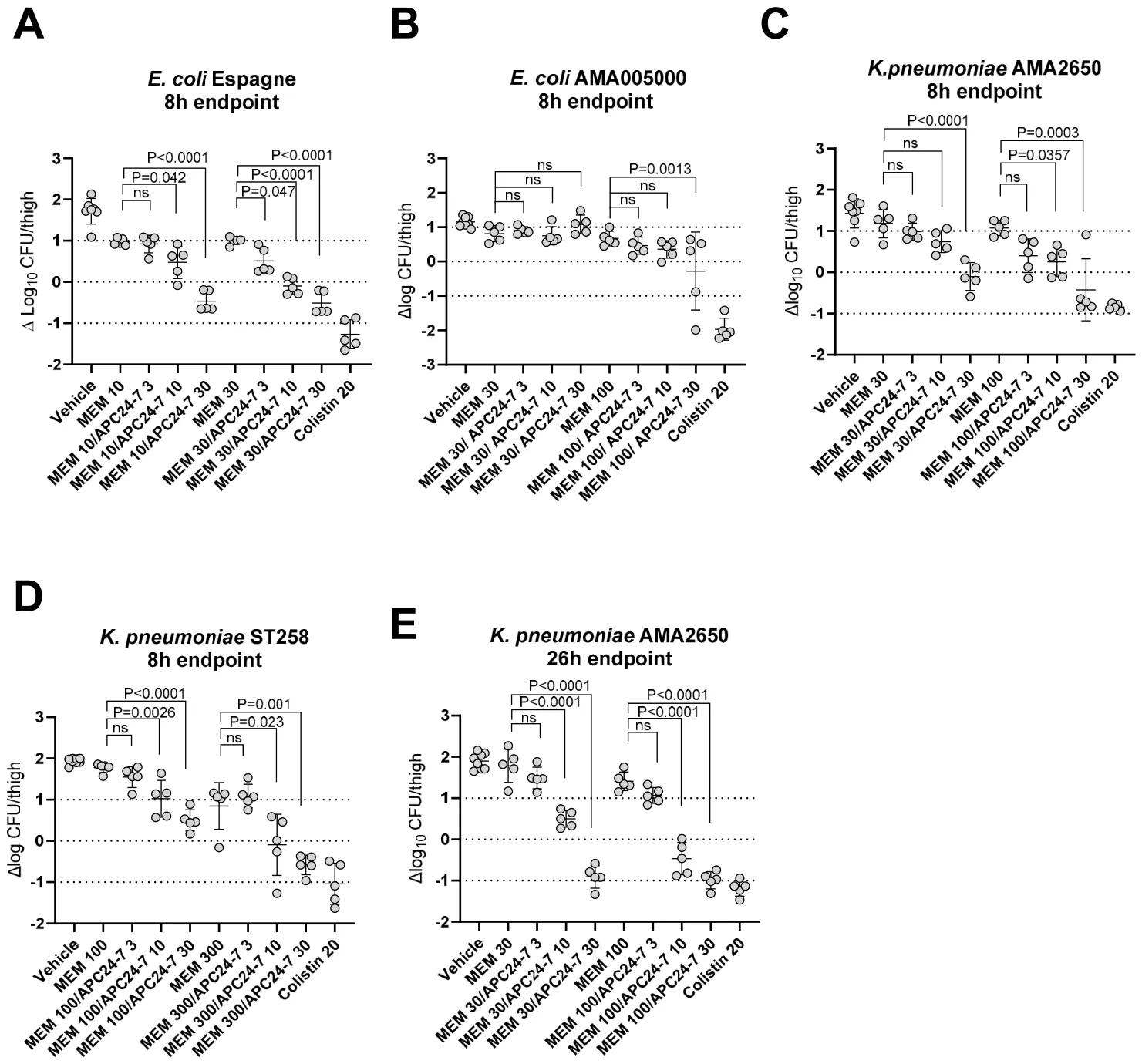
In vivo effect of meropenem/APC24-7 combination treatment. Bacterial growth at 8 h after inoculation, relative to the start-of-treatment baseline (approximately 6 log_10_ CFU for all isolates), is shown after two doses administered at 2 and 6 h after inoculation in mice infected with carbapenem-resistant isolates: (**A**) *E. coli* Espagne, (**B**) *E. coli* AMA005000, (**C**) *K. pneumoniae* AMA2650, and (**D**) *K. pneumoniae* ST258. (**E**) For *K. pneumoniae* AMA2650, an additional experiment assessed bacterial burden at 26 h after Q4h dosing initiated 2 h after inoculation. Colistin was administered Q12h as a positive control. Statistical comparisons were made against the corresponding meropenem monotherapy group using ANOVA with Sidak-adjusted multiple comparisons. Individual doses are indicated in mg/kg (e.g., MEM 100 = meropenem 100 mg/kg Q4h).

For the 8 h experiments, two subtherapeutic meropenem dose levels were evaluated in combination with APC24-7 at 3, 10, or 30 mg/kg. Treatments were administered at 2 and 6 h after inoculation (Figure 6A–D). APC24-7 increased meropenem activity in a dose-dependent manner against all four isolates. Colistin at 20 mg/kg QD (once daily) or BID (twice daily), estimated to provide clinically relevant exposure over the duration of the experiments [46,47], served as a positive control treatment.

In mice infected with *E. coli* Espagne (VIM-1; MEM MIC = 32 mg/L), 30 mg/kg APC24-7 reduced the mean bacterial burden by ≥1 log10 CFU relative to meropenem 10 mg/kg monotherapy, bringing the bacterial burden back to the start-of-treatment baseline (Figure 6A). Increasing the meropenem dose to 30 mg/kg enabled 10 mg/kg APC24-7 to reduce the mean bacterial load to baseline (Figure 6A). The largest mean reduction relative to meropenem monotherapy was 1.512 log_10_ CFU (95% CI, 1.025–1.999) and was observed with meropenem 30 mg/kg plus APC24-7 30 mg/kg (Table 4).

*E. coli* AMA005000 (NDM-5, OXA-48, CTX-M-3, TEM-1B; MEM MIC = 128 mg/L) was less responsive to APC24-7 in vivo. Only the combination of meropenem 100 mg/kg and APC24-7 30 mg/kg reduced the mean bacterial burden to baseline (Figure 6B). This regimen produced a mean reduction of 0.946 log_10_ CFU (95% CI, 0.3293–1.563) relative to meropenem monotherapy (Table 4).

*K. pneumoniae* isolates AMA2650 (KPC-3, SHV-62 *, TEM-1A *; MEM MIC = 128 mg/L) and ST258 (KPC-2, OXA-9-like, SHV-5 *, TEM-1A *; MEM MIC = 64 mg/L) were both highly responsive to APC24-7. Depending on meropenem exposure, APC24-7 doses of 10 or 30 mg/kg reduced the mean bacterial burden below baseline (Figure 6 C,D), and the combination was as efficacious as colistin treatment. For ST258, the largest mean reduction relative to meropenem monotherapy was 1.428 log_10_ CFU (95% CI, 0.6054–2.251) and was observed with meropenem 300 mg/kg plus APC24-7 30 mg/kg (Table 4). For AMA2650, the largest mean reduction was 1.5 log_10_ CFU (95% CI, 0.7272–2.273) and was observed with meropenem 100 mg/kg plus APC24-7 30 mg/kg (Table 4). A subsequent 26 h experiment with *K. pneumoniae* AMA2650 evaluated repeated Q4h administration of the combination. The most active combination regimen reduced the bacterial burden to approximately 1 log_10_ CFU below the start-of-treatment baseline (Figure 6E). With meropenem 30 mg/kg plus APC24-7 30 mg/kg, the mean reduction relative to the corresponding meropenem monotherapy group was 2.692 log_10_ CFU (95% CI, 2.210–3.174) (Table 4), demonstrating that the combination effect could be maintained during repeated dosing.

## 3. Discussion

Carbapenems, including meropenem, remain important last-resort therapies for severe infections caused by Gram-negative bacteria. The increasing prevalence of β-lactamase-mediated resistance therefore poses a major therapeutic challenge [11,48]. Broad-spectrum BLIs capable of inhibiting both SBLs and MBLs could therefore extend the utility of existing β-lactam antibiotics.

APC24-7 was previously described as a hybrid BLI with activity against both SBLs and MBLs. It showed greater activity against NDM-9 and IMP-26 than taniborbactam, whereas activity against OXA-48 was limited. Among several β-lactam partners evaluated, meropenem showed the most consistent activity in combination with APC24-7 [43]. The present study extends this earlier characterization by evaluating the meropenem/APC24-7 combination across a diverse panel of meropenem-resistant *E. coli* and *K. pneumoniae* isolates and by providing in vivo proof of concept in a murine infection model.

The present findings provide preclinical proof of concept for APC24-7 as a dual MBL/SBL inhibitor capable of potentiating meropenem across multiple β-lactamase backgrounds, although the magnitude of potentiation was strongly isolate- and concentration-dependent.

In checkerboard MIC assays, APC24-7 potentiated meropenem against clinical isolates of *K. pneumoniae* and *E. coli* with high meropenem MICs ranging from 16 to >1024 mg/L and harboring a broad range of β-lactamase genes. Responses varied substantially among isolates. For most *K. pneumoniae* isolates with very high meropenem MICs (≥256 mg/L), APC24-7 reduced the meropenem MIC several-fold but did not lower it to the CLSI susceptible breakpoint, even at 64 mg/L.

Three of the 31 *K. pneumoniae* isolates (9.7%) showed no response to APC24-7, even at 64 mg/L. Consistent with the previously reported limited activity of APC24-7 against OXA-48 [43], all three unresponsive isolates were OXA-48-positive. However, other OXA-48-positive isolates in the present study showed substantial reductions in meropenem MICs, indicating that the presence of OXA-48 alone cannot explain the observed phenotype. Because many isolates carried multiple β-lactamase genes, the contribution of individual enzymes also cannot be inferred directly from these experiments. The observed variability may reflect additional resistance mechanisms, including altered outer-membrane permeability, efflux, target modification, and regulatory pathways involved in β-lactamase expression and sensing [12,49]. These mechanisms were not systematically investigated in the present study and should be addressed in future work.

Time-kill experiments provided further evidence that APC24-7 can substantially potentiate meropenem activity. In all nine selected isolates, active combination conditions produced a ≥3 log10 CFU/mL reduction from the initial bacterial count within 3–5 h. Although incomplete killing or regrowth was observed at later time points under some treatment conditions, regrowth occurred predominantly with meropenem monotherapy and less frequently under active combination conditions, suggesting that APC24-7 may limit regrowth under the conditions tested. Preliminary susceptibility testing of colonies recovered following meropenem monotherapy suggested the selection of less susceptible subpopulations; however, further genomic and phenotypic characterization is required to determine the underlying mechanism.

It is also important to note that zMIC values derived from the first 3–5 h represent early-phase net-growth pharmacodynamics and should not be compared directly with the conventional 16–20 h MIC values in Table 1 and Table 2, which incorporate later bacterial growth or regrowth and differ conceptually from zMIC. Differences between these measures therefore do not necessarily indicate a decline in inhibitor activity. For *E. coli* IHMA997800, increasing APC24-7 concentrations above 32 mg/L produced little additional reduction in the minimum fitted net growth rate, although the meropenem concentration–response relationship remained concentration-dependent. Together, these isolate-specific observations may help explain the variable effect of APC24-7 on meropenem activity across the broader isolate panel.

The in vivo experiments further supported APC24-7 as a BLI. In the murine thigh infection model, APC24-7 increased meropenem activity against four highly meropenem-resistant clinical isolates at dose levels at which meropenem monotherapy had little or no effect on bacterial burden. For this proof-of-concept study, isolates were selected based on their in vitro responsiveness to APC24-7, including reduction in the meropenem MIC into the CLSI susceptible range. Nevertheless, the magnitude of the in vivo effect varied among isolates. In particular, the combination was less effective against *E. coli* AMA005000 (NDM-5, OXA-48), despite similar responsiveness in checkerboard and time-kill assays. This discrepancy may reflect the contribution of OXA-48, against which APC24-7 has limited activity, or differences in β-lactamase activity and other resistance mechanisms between in vitro and in vivo conditions.

The PK study was designed to support these murine proof-of-concept experiments rather than to define a clinically translatable dosing regimen. APC24-7 was administered subcutaneously for experimental convenience, and the less-than-dose-proportional exposure and delayed Tmax observed at the highest dose may reflect dose-dependent absorption, formulation-related limitations, or nonlinear systemic disposition. Because these possibilities cannot be distinguished from the present SC data, intravenous PK and formulation studies will be required to establish absolute bioavailability and systemic disposition. More importantly, the APC24-7 exposure required for sustained potentiation of meropenem has not yet been defined. For other BLIs, PK/PD activity has been related to either time above an inhibitor threshold concentration or AUC-based indices, depending on the inhibitor and β-lactam partner [50]. Translational development of APC24-7 will therefore require characterization of the joint meropenem/APC24-7 exposure–response relationship across multiple isolates and resistance backgrounds, preferably using clinically relevant or human-simulated meropenem exposures [51]. Such studies should also include isolates with very high meropenem MICs and isolates for which APC24-7 does not reduce the meropenem MIC to the susceptible range. These data, together with further safety, PK, and formulation studies, will be needed to define an appropriate clinical dose and dosing ratio. The advancement of related molecules, such as APC148, into early clinical evaluation nevertheless supports the feasibility of this inhibitor strategy.

Overall, our findings provide preclinical proof of concept and suggest that meropenem combined with APC24-7 may represent a complementary β-lactamase-inhibition strategy, particularly for resistance backgrounds dominated by KPC and selected MBLs, for which current therapeutic options remain limited.

## 4. Materials and Methods

### 4.1. Bacterial Isolates and Compounds

A panel of clinical isolates representing a broad range of β-lactamase genes was obtained from Statens Serum Institut (SSI) and the CDC antimicrobial resistance collection, with additional isolates provided by collaborating laboratories. ATCC 25922, ATCC BAA-1705, and NCTC 13443 were included as quality-control strains. Detailed information on the bacterial panel is provided in Table S1. Isolates were selected to represent a diverse range of clinically relevant β-lactamase genes and meropenem resistance levels in *E. coli* and *K. pneumoniae*. Meropenem for injection was purchased from STADA. APC24-7 was synthesized by Kappa Solutions AS, which was subsequently acquired by Balchem Corporation, Montvale, NJ, USA.

### 4.2. Genome Analysis

Short-read whole-genome sequencing (WGS) was performed as part of the national surveillance programme. Genomic DNA was extracted using the MagNA Pure 96 DNA and Viral NA Multi-Sample Kit (Life Technologies, Carlsbad, CA, USA), and sequencing libraries were prepared using the Illumina Nextera XT DNA Library Preparation Kit (Illumina, San Diego, CA, USA). Libraries were sequenced on an Illumina NextSeq platform, generating 2 × 150 bp paired-end reads. Raw sequencing reads were quality controlled and processed using the in-house Bifrost pipeline (https://github.com/ssi-dk/bifrost, accessed on 7 October 2026). Draft genome assemblies were generated using SKESA, and sequencing runs with an average genome coverage below 30× were excluded. Resistance genes mapped with coverage and identity below 95% were excluded, whereas genes mapped with coverage and identity of at least 95% but less than 100% were marked with an asterisk (*).

Multi-locus sequence types and antimicrobial resistance genes were extracted as part of the Bifrost pipeline based on databases obtained from pubMLST.org and ResFinder V4.7.2, respectively. Cluster types (CTs) were inferred using Ridom MBioSeq Ridom Typer v12.0. Information regarding the genomic data for ATCC (American Type Culture Collection) and CDC (Centers for Disease Control and Prevention) isolates can be found on the respective websites.

### 4.3. Determination of Minimum Inhibitory Concentration (MIC)

The minimum inhibitory concentrations (MICs) of meropenem and APC24-7 were determined by broth microdilution in accordance with Clinical and Laboratory Standards Institute (CLSI) guidelines [44].

In brief, serial twofold dilutions of meropenem or APC24-7 were prepared in cation-adjusted Mueller–Hinton broth in 96-well plates. Meropenem was tested from 1 to 1024 mg/L and APC24-7 from 1 to 64 mg/L. Each well was inoculated with the standard inoculum recommended by CLSI. Appropriate ATCC strains were included as quality-control (QC) strains. *E. coli* ATCC 25922 served as the meropenem MIC control and was not included in the other assays. MIC determinations were performed in duplicate.

### 4.4. Checkerboard MIC Assays

Checkerboard MIC assays were performed in 96-well microtiter plates using the method described by Bellio et al. (2021) [52], with minor modifications. Plates were arranged with decreasing meropenem concentrations along the *x*-axis and APC24-7 concentrations along the *y*-axis. Meropenem was tested from 0.6 to 64 mg/L and APC24-7 from 1 to 64 mg/L. Each well received 50 µL of bacterial suspension at approximately 10^6^ CFU/mL together with the antibiotic and inhibitor. Plates were incubated at 35 ± 2 °C for 18 h. Each experiment was performed in duplicate. Potentiation of meropenem by APC24-7 was defined as a ≥4-fold reduction in meropenem MIC relative to meropenem alone [52].

### 4.5. Time-Kill Experiments

Nine clinical isolates—*E. coli* IHMA997800, *E. coli* Espagne, *E. coli* AMA005000, *E. coli* 149, *K. pneumoniae* ST258, *K. pneumoniae* B68-1, *K. pneumoniae* AMA4817, *K. pneumoniae* 1149, and *K. pneumoniae* AMA2650—representing different β-lactamase genes were selected for time-kill assays. Assays were performed in duplicate according to the Clinical and Laboratory Standards Institute (CLSI) document M26-A [45] with minor modifications. Briefly, 96-well plates were inoculated with bacterial suspensions at approximately 10^5^ CFU/mL. Meropenem was tested at 0.25, 1, 4, and 16 mg/L and at the MIC of each strain, both alone and in combination with a fixed APC24-7 concentration of 32 mg/L. Plates were incubated at 35 ± 2 °C with shaking for 24 h, and samples were collected at 0, 1, 3, 5, 8, and 24 h for CFU determination. Time-kill curves were generated by plotting mean log_10_ CFU/mL against time to compare 24 h bacterial killing with meropenem monotherapy and the meropenem/APC24-7 combination.

### 4.6. Fitting of an Emax-Type of Bacterial Growth-Rate Model to Time-Kill Data

Equation (1) was used to estimate the initial net growth rate *µ* based on the mean slope defined by the first few time points of CFU data (from *t* = 0 to *t* = Δ*t*) for each curve, i.e., for each tested concentration *c* of meropenem (for fixed concentration of APC24-7). Values of 3 and 5 h were used for Δ*t* in all *µ* estimates.

(1) $$\mu =(\ln\left({CFU}_{\Delta t}\right)-\ln\left({CFU}_{0}\right))∕\Delta t$$

An Emax-type of net growth rate model (Equations (2) and (3)) was then fitted to the *µ*(*c*) data points:

(2) $$\mu =\mu_{\max}-(\mu_{\max}-\mu_{\min})∕(1+{\left({EC}_{50}∕c\right)}^{\varkappa })\;for\;c\;>\;0$$

(3) $$\mu =\mu_{\max}\;for\;c=0$$

where *ϰ* is the negative slope of the sigmoid curve at meropenem concentration *c* = *EC*_50_.

In the parameter estimation procedure, *μ*_max_ was fixed to the value found for the growth control curve (*c* = 0 mg/L), which left three parameters free to be estimated: *μ*_min_, *EC*_50_ and *ϰ*.

If *μ*_min_ < 0, a pharmacodynamic MIC, *zMIC*, can furthermore be defined as the concentration of the antibiotics at which *μ* = 0. The value of *zMIC* is closely related to *EC*_50_ and can be derived from Equation (2). All parameter estimation was done with the TimKil program in the PKPDsim software package [53].

### 4.7. Bacterial Inoculum for In Vivo Studies

*K. pneumoniae* and *E. coli* strains were grown overnight on 5% horse blood agar (SSI Diagnostica, Copenhagen, Denmark). Approximately 1 h before inoculation, single colonies were suspended in sterile 0.9% saline to an optical density of 0.13 at 540 nm, corresponding to approximately 2 × 10^8^ CFU/mL. The suspension was then diluted in 0.9% saline to 2 × 10^7^ CFU/mL. Each mouse received 0.05 mL intramuscularly in the left thigh, corresponding to approximately 10^6^ CFU. For each experiment, the inoculum concentration was verified by preparing tenfold serial dilutions in 0.9% saline, plating 20 µL on 5% blood agar, and counting colonies after overnight incubation at 35 ± 2 °C in ambient air.

### 4.8. Mice and Housing

Outbred female HsdWin:NMRI mice, 26–30 g (Inotiv, Kreuzelweg, The Netherlands), were housed at the animal facility at Statens Serum Institut. Mice were acclimatized for 4–7 days before experimentation. Animals were allocated to experimental groups using a serpentine allocation procedure. Body weights were assessed before allocation, and mice were distributed to achieve comparable body-weight distributions across groups. No random-number-generated allocation sequence was used. Room temperature and relative humidity were maintained at 22 ± 2 °C and 55 ± 10%, respectively. Mice were housed in individually ventilated type 3 Macrolone cages with Tapvei bedding. The air exchange rate was approximately 8–12 air changes per hour in the room and 70–73 air changes per hour inside the cages. A 12 h light/12 h dark cycle was used (lights on from 6 a.m. to 6 p.m.). Mice had ad libitum access to domestic-quality drinking water and food (Teklad Global Diet 2916C, Inotiv, Køge Kreuzelweg, The Netherlands); peanuts and sunflower seeds (Køge Korn A/S, Køge, Denmark) were provided occasionally. Enviro-Dri nesting material and cardboard houses (Bio-Serv) were provided as enrichment. Studies were ethically reviewed and conducted in accordance with European Directive 2010/63/EEC and Danish license No. 2021-15-0201-01046.

### 4.9. Pharmacokinetic Study and Bioanalysis

Pharmacokinetic studies were performed in infected female neutropenic NMRI mice 16 h after intramuscular infection, as described below. APC24-7 was administered SC at 3, 10, or 30 mg/kg. At each sampling time, plasma was collected from three mice (*n* = 3), and concentrations are reported as mean ± SE. Plasma samples were collected at 5, 15, 30, 60, and 120 min after dosing and at approximately 160 min, when some mice met the humane endpoint because of infection. The in-life phase was conducted at SSI, and bioanalysis was performed by Lablytica (Uppsala, Sweden). Separation was performed by reversed-phase gradient high-performance liquid chromatography (LC) using a C18 analytical column and gradient elution with 0.1% formic acid in water and acetonitrile. Detection was performed by mass spectrometry (MS) using positive-ion spray ionization. The MS settings included an ion-spray voltage of 1.0 kV, a cone-gas flow of 30 L/h, and a desolvation-gas flow of 1000 L/h. PK parameters were calculated by noncompartmental analysis using the initESTIM program in the PKPDsim software package [53].

### 4.10. Murine Thigh Infection Model

A previously described neutropenic murine thigh infection model was used for all experiments [36]. In brief, female NMRI mice were rendered neutropenic by cyclophosphamide pretreatment at 150 and 100 mg/kg IP on days −4 and −1 relative to inoculation, respectively. Mice were then inoculated intramuscularly in the left thigh with approximately 6 log_10_ CFU. Treatment was initiated 2 h after inoculation and repeated Q4h. Endpoints were 8 h (after two doses) or 26 h (after six doses) after inoculation. Treatment administration was not blinded; however, personnel performing tissue processing and CFU enumeration were unaware of treatment allocation. The primary efficacy outcome was bacterial burden in the infected thigh, expressed as log_10_ CFU/thigh at the terminal time point. The individual mouse was the experimental unit. A separate untreated baseline group (*n* = 5) was euthanized 2 h after inoculation, immediately before treatment initiation, to determine the start-of-treatment bacterial burden. Treatment groups comprised five mice (*n* = 5), and vehicle-control groups comprised eight mice (*n* = 8). No formal a priori power calculation was performed; group sizes were selected based on historical variability and previous experience with the neutropenic thigh infection model. Prespecified technical exclusion criteria included unsuccessful inoculation or dosing. No animals were excluded from the analyses.

Bacterial isolates were selected based on their ability to proliferate in vivo, their limited response to meropenem monotherapy, and their in vitro response to the meropenem/APC24-7 combination.

Mice were monitored for clinical signs of infection throughout the study. In the 8 h experiments, clinical scores were mild across all groups. In the 26 h experiment, scores were mild to moderate in vehicle control mice, and no mice met the humane endpoint. Meropenem was supplied as STADA 500 mg powder for injection. Meropenem and APC24-7 were mixed and administered as a single subcutaneous injection. All injectable preparations were kept refrigerated and protected from light.

### 4.11. Statistical Analysis

The individual mouse was the experimental unit. CFU counts were log10-transformed before analysis. Homogeneity of variance was assessed using the Brown–Forsythe test. Treatment groups were compared by one-way ANOVA followed by Sidak-adjusted multiple comparisons with the corresponding meropenem monotherapy group. For graphical presentation, bacterial burdens are expressed as the change from the mean 2 h start-of-treatment baseline. Statistical comparisons were based on endpoint Δlog10 CFU/thigh values and are reported with 95% confidence intervals and adjusted *p* values. Analyses were performed using GraphPad Prism 11.0.0 for Windows.

## 5. Conclusions

APC24-7 potentiated meropenem activity in vitro and in a neutropenic murine thigh infection model against selected carbapenem-resistant *E. coli* and *K. pneumoniae* isolates. The magnitude of potentiation was isolate- and concentration-dependent, with variable activity among OXA-48-harboring isolates. These in vivo studies provide proof of concept rather than a clinically translatable dosing regimen, and the APC24-7 exposure required to maintain sufficient β-lactamase inhibition remains to be established. Further PK/PD characterization using clinically relevant meropenem exposures and a broader range of resistance backgrounds is warranted.

## Figures and Tables

**Table 1:** Meropenem susceptibility profiles of *K. pneumoniae* clinical isolates, alone and in combination with APC24-7.

Isolate ID	β-Lactamase (s)	Meropenem MIC (mg/L)					
		-	APC24-7 (mg/L)				
			4	8	16	32	64
76	VIM-1, CTX-M-15, CTX-M-9, OXA-1	32	0.25	0.25	≤0.06	≤0.06	≤0.06
AMA2650	KPC-3, SHV-62 *, CTX-M-15, TEM-1A *, OXA-9 *	128	0.5	0.5	0.25	0.25	0.125
DIH	VIM-19, SHV-172, TEM-1B, CMY-4, CTX-M-3	64	1	0.5	0.125	≤0.06	≤0.06
AMA2119	KPC-3, SHV-28 *, CTX-M-15, TEM-209 *, OXA-9 *, OXA-1 *	128	4	2	0.5	0.125	0.125
135	VIM-1, SHV-12, TEM-1A, OXA-9	32	4	2	≤0.06	≤0.06	≤0.06
160	OXA-48, SHV-11	16	4	2	≤0.06	≤0.06	≤0.06
ATCCBAA1705	KPC-2	64	4	2	0.5	0.25	0.125
34	IMP-4, SHV-11, TEM-1	16	8	8	8	2	≤0.06
AMA004435	OXA-48, SHV-26 *	128	16	4	1	0.5	0.125
B68-1	NDM-1, AmpH, CTX-M-15, CMY, OXA-9, SHV-33, TEM-168	128	16	4	1	≤0.06	≤0.06
ST258	KPC-2, OXA-9 *, SHV-5 *, TEM-1A *	64	16	16	2	0.5	0.125
SECURE ST147	NDM-1, KPC-2	64	16	8	4	0.5	0.25
AMA877	KPC-3, SHV-182 *, TEM-1B, OXA-9 *, OXA-1	128	16	16	4	0.25	0.25
80	IMP-4, OKP-B-21, OXA-1, SFO-1, TEM-1	16	16	16	16	4	0.125
AMA004417	VIM-1, KPC-2, SHV-99, TEM-1B, OXA-10	1024	32	16	8	0.5	0.125
850	OXA-1, OXA-48, SHV-11, TEM-1	64–128	32	32	32	32	32
1149	IMP-18, KPC-45, OXA-1, OXA-2, SHV-1, CTX-M-15	32	32	32	32	32	≤0.06
AMA004817	NDM-9, SHV-28, CTX-M-15, OXA-1, TEM-1B	128	>64	>64	16	0.5	≤0.06
NCTC13443	NDM-1, CMY-4, CTX-M-15, SHV-106, SHV-28, OXA-1, DHA-24, DHA-1, TEM1-B, TEM1-A, SHV-1A, SHV-185, SHV-11, SHV-70, SHV-69, SHV-31, SHV-25, SHV-13, OXA-9	512	>64	>64	>64	4	1
40	VIM-27, CTX-M-15, OXA-1, SHV-11	1024	>64	>64	>64	32	1
AMA003821	KPC-2, SHV-12	1024	>64	>64	>64	64	16
AMA1348	KPC-2, SHV-187 *, TEM-1A *, OXA-9 *	1024	>64	>64	64	16	8
CPO20170084	NDM-1, OXA-48, SHV-28 *, CTX-M-15 *, OXA-9, TEM-1A	256	>64	>64	64	32	16
AMA004208	NDM-4, OXA-181, SHV-1, TEM-1B	1024	>64	>64	>64	32	8
AMA004950	NDM-5, OXA-232, SHV-182 *, CTX-M-15, TEM-1B	>1024	>64	>64	>64	64	32
AMA004443	KPC-2, NDM-1, CTX-M-15, SHV-12	1024	>64	>64	>64	8	2
AMA1498	KPC-3, SHV-28 *	1024	>64	>64	64	16	8
1158	NDM-5, VIM-2, CTX-M-15, OXA-1, OXA-232, SHV-11, TEM-1	512	>64	>64	>64	64	16
ST147	OXA-48, SHV-67 *, VEB-8, CTX-M-15, CMY-2, OXA-1, TEM-1B	64	64	64	64	64	64
AMA005047	NDM-5, OXA-48, SHV-67 *, CTX-M-15, CTX-M-14b, TEM-1B *, OXA-9 *	256	>64	>64	>64	>64	>64
556	CTX-M-15, OXA-48, OXA-9, SHV-100, TEM-1A	16–32	16	16	16	16	16

Grey, no change in MIC; Green, susceptible (≤1 mg/L); Yellow, intermediate (MIC = 2 mg/L); Red, resistant, (MIC ≥ 4 mg/L). * Indicates mapping to the reference gene with both coverage and identity ≥95% but <100%. “-“ means no APC24-7 was added

**Table 2:** Meropenem susceptibility profiles of *E. coli* clinical isolates, alone and in combination with APC24-7.

Isolate ID	β-Lactamase (s)	Meropenem MIC (mg/L)					
		-	APC24-7 (mg/L)				
			4	8	16	32	64
AMA004326	KPC-3, OXA-9 *, TEM-1A *	32	0.125	0.125	0.125	0.125	0.06
AMA003244	NDM-5, OXA-232, CTX-M-15, TEM-1B	64	1	0.5	0.5	0.5	0.125
149	NDM-7, CMY-42	64	4	2	2	2	1
AMA817	NDM-1, CMY-6, TEM-1C, OXA-1	32	8	2	1	0.125	0.06
AMA005000	NDM-5, OXA-48, CTX-M-3, TEM-1B	128	8	4	2	1	0.5
AMA004995	NDM-5, OXA-181, CMY-42	256	8	8	2	1	0.25
1162	NDM-5, CTX-M-15, OXA-48	256	16	8	8	2	0.5
AMA005098	KPC-3, NDM-5, SHV-1, CTX-M-15, CMY-145	256	32	8	4	0.5	0.25
IR57	NDM-1, CTX-M-15, TEM-198, AmpC, OXA-1, FonA-2	256	32	16	2	1	0.125
IHMA997800	NDM-5, CTX-M-15, TEM-1-B, CMY-2	256	32	32	8	4	2
AMA003170	NDM-5, OXA-181, CTX-M-15, CMY-2, OXA-1, TEM-1B	32	32	16	8	4	1
CPO20180141	NDM-5, OXA-244, CTX-M-15, OXA-1	32	32	8	4	1	0.125
AMA005043	NDM-5, OXA-244	128	64	64	16	4	0.25
Espagne	VIM-1, TEM-1B	32	64	64	16	1	0.25
162	NDM-7, CTX-M-15, TEM-1B	128	>64	64	32	8	0.125
OM20	NDM-1, CTX-M-15, TEM-1	128	128	64	16	1	0.25
IHMA997577	NDM-5, CTX-M-15, TEM-1-B	256	256	64	32	2	0.25
ATCC 25922	-	0.03	--	--	--	--	--

Grey, no change in MIC; Green, susceptible (≤1 mg/L); Yellow, intermediate (MIC = 2 mg/L); Red, resistant, (MIC ≥ 4 mg/L). * Indicates mapping to the reference gene with both coverage and identity ≥95% but <100%. “-“ means no APC24-7 was added.

**Table 3:** Plasma pharmacokinetic parameters from noncompartmental analysis of APC24-7 in thigh-infected female NMRI mice following a subcutaneous dose. Because absolute bioavailability (F) was not determined, clearance and volume of distribution are reported as apparent parameters (CL/w and Vss/w, respectively).

APC24-7 Dose	T½ (h)	Tmax [h]	Cmax [mg/L]	AUCinf [h∙mg/L]	Vss/w [L/kg]	CL/w [L/ (h∙kg)]	Kel’ [/h]
30 mg/kg SC	0.77	1.0	26	51	0.80	0.58	0.73
10 mg/kg SC	0.79	0.25	14	19	0.65	0.53	0.82
3 mg/kg SC	0.66	0.25	7.7	8.5	0.37	0.35	0.95

**Table 4:** Statistical summary of the most effective dose combinations in the in vivo efficacy experiments.

Isolate ID	Endpoint	Group Comparison	Mean Diff	95% CI of Diff	Adjusted *p* Value
*E.c.* Espagne	8 h	MEM 30 vs. MEM 30/APC24-7 30	1.512	1.025 to 1.999	<0.0001
*E.c.* AMA005000	8 h	MEM 100 vs. MEM 100/APC24-7 30	0.946	0.3293 to 1.563	0.0013
*K.p.* AMA2650	8 h	MEM 100 vs. MEM 100/APC24-7 30	1.5	0.7272 to 2.273	0.0003
*K.p.* ST258	8 h	MEM 300 vs. MEM 300/APC24-7 30	1.428	0.6054 to 2.251	0.001
*K.p.* AMA2650	26 h	MEM 30 vs. MEM 30/APC24-7 30	2.692	2.210 to 3.174	<0.0001

## Data Availability

The original contributions presented in this study are included in the article and Supplementary Materials. Information regarding bacterial genomes/accession information can also be found in Supplementary Materials. Further inquiries can be directed to the corresponding author.

## Supplementary Materials

The following supporting information can be downloaded at https://www.mdpi.com/article/10.3390/antibiotics15101011/s1. Figure S1: Time-kill curves of meropenem monotherapy against selected *K. pneumoniae* and *E. coli* isolates; Table S1: Characteristics and antimicrobial resistance genotypes of the clinical and reference isolates included in the study.

## Abbreviations

The following abbreviations are used in this manuscript:

BL	β-lactam antibiotic
BLI	β-lactamase inhibitor
SBL	serine β-lactamase
MBL	metallo-β-lactamase
CRE	carbapenem-resistant Enterobacterales
MEM	meropenem
CZA	ceftazidime-avibactam
IMR	imipenem-relebactam
PBP	penicillin-binding protein
MIC	minimum inhibitory concentration
zMIC	zero-net-growth MIC
CFU	colony-forming units
PK	pharmacokinetics
PK/PD	pharmacokinetics/pharmacodynamics
Cmax	maximum plasma concentration
AUCinf	area under the concentration–time curve extrapolated to infinity
Tmax	time to maximum plasma concentration
T½	terminal half-life
Vss/w	steady-state volume of distribution normalized to body weight
CL/w	clearance normalized to body weight
kel′	terminal elimination rate constant
CT	cluster type
SC	subcutaneous
QD	once daily
BID	twice daily
LOQ	limit of quantification
Q4h	every 4 h
Q12h	every 12 h
WGS	whole-genome sequencing
CLSI	Clinical and Laboratory Standards Institute
WHO	World Health Organization
ATCC	American Type Culture Collection
CDC	Centers for Disease Control and Prevention
LC	liquid chromatography
MS	mass spectrometry
