# Supplementary material for: In Vitro and In Vivo Efficacy of the Broad-Spectrum β-Lactamase Inhibitor APC24-7 in Combination with Meropenem Against Carbapenem-Resistant Enterobacterales
Source: Antibiotics (Basel). 2026 Oct 12;15(10):1011. doi: 10.3390/antibiotics15101011 (PMC13700718; doi:10.3390/antibiotics15101011)
Supplement: Supplementary file 1 [file antibiotics-15-01011-s001.zip › Figure S1. Meropenem monotherapy time-kill curves_revised.pdf]

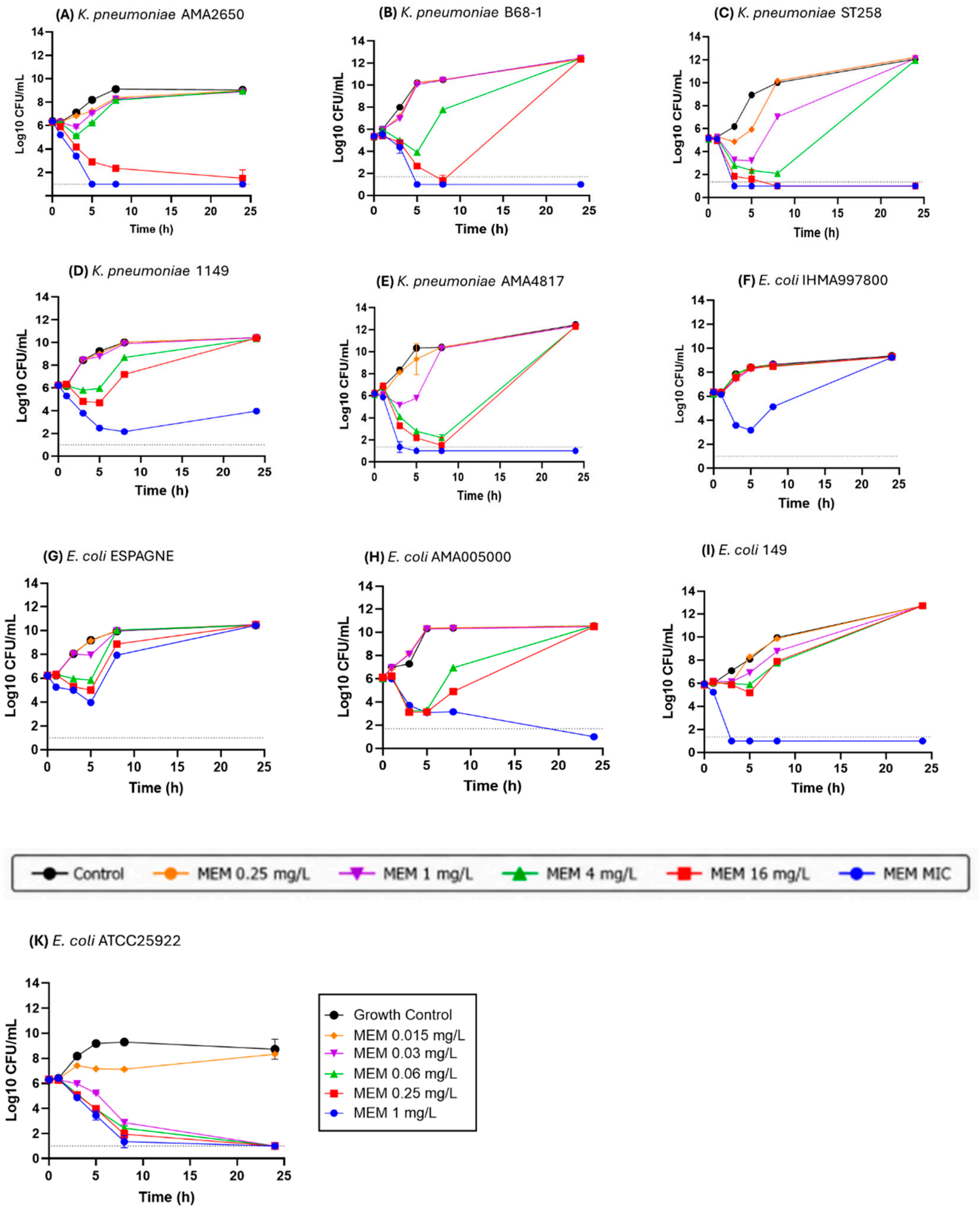

Figure S1. For meropenem-resistant clinical isolates (MEM MIC ≥ 32 mg/L): (A) *K. pneumoniae* AMA2650, (B) *K. pneumoniae* B68-1, (C) *K. pneumoniae* ST258, (D) *K. pneumoniae* 1149, (E) *K. pneumoniae* AMA4817, (F) *E. coli* IHMA997800, (G) *E. coli* Espagne, (H) *E. coli* AMA005000, (I) *E. coli* 149 (K) and *E. coli* ATCC25922 (control), treated with meropenem alone at MIC value, 0.25, 1, 4 or 16 mg/L. Growth controls (drug free medium) were included in all experiments. Growth controls (drug-free medium) were included in all experiments. The dotted line in each graph indicates the lower limit of detection. Data are shown as mean ± SD $\log_{10}$ CFU/mL from duplicate measurements at each time point.
